# Ameliorative effects of vitamin C and garlic (*Allium sativum*) on nifedipine-induced reproductive dysfunction in a male albino rat model

**DOI:** 10.3389/fvets.2025.1725725

**Published:** 2026-01-29

**Authors:** Ugochinyere J. Njoga, Kenneth O. Anya, Samuel O. Ekere, Faith. N. Amune, Lawrence U. Eze, Mark E. Awachie, Loveth C. Ojimba, Izuchukwu S. Ochiogu, Emmanuel O. Njoga

**Affiliations:** 1 Department of Veterinary Obstetrics and Reproductive Diseases, Faculty of Veterinary Medicine, University of Nigeria, Nsukka, Nigeria; 2 Department of Veterinary Physiology and Pharmacology, Faculty of Veterinary Medicine, University of Nigeria, Nsukka, Nigeria; 3 Department of Agricultural and Animal Health, College of Agriculture Environmental Sciences, University of South Africa, Johannesburg, South Africa; 4 Department of Public Health and Preventive Medicine, Faculty of Veterinary Medicine, University of Nigeria, Nsukka, Nigeria

**Keywords:** ameliorative effects, garlic, male albino rats, nifedipine, sperm cells, vitamin C

## Abstract

**Introduction:**

Nifedipine, a calcium channel blocker widely prescribed for hypertension, has been implicated in oxidative stress–mediated reproductive toxicity. This study investigated the ameliorative effects of vitamin C and garlic (*Allium sativum*) on nifedipine-induced reproductive dysfunction in a male albino rat model.

**Methods:**

Twenty-five adult male rats were randomized into control and treatment groups, receiving nifedipine (0.571 mg/kg) alone or in combination with vitamin C (200 mg/kg) and/or garlic extract (200 mg/kg) for 40 days. Reproductive parameters and histopathological changes in the testes and epididymis were assessed.

**Results:**

Nifedipine administration significantly reduced sperm parameters compared to control-sperm motility (38.9 ± 2.53 vs. 83.25 ± 1.65 in controls, *P* ≤ 0.05), viability (88.03 ± 1.87 vs. 94.00 ± 0.98), and normal morphology (80.75 ± 0.85 vs. 89.5 ± 0.65), while co-administration of vitamin C and vitamin C + garlic combination slightly mitigated these effects, improving sperm motility to 41.25 ± 6.95 and 45.00 ± 7.5, respectively. The combined treatment produced the most profound improvement, restoring sperm motility (6.1 % increase), viability (3.15 % increase, close to control values), and normal sperm (4% ameliorative effect, *P* ≤ 0.05). Histopathological analysis revealed substantial preservation of testicular and epididymal integrity in the vitamin C and less in the combination (vitamin C + garlic) and garlic treatment groups.

**Conclusion:**

Nifedipine caused significant impairments in sperm quality and histological integrity. Vitamin C and garlic combination produced a statistically significant improvement in normal sperm morphology, with other parameters showing non-significant numerical increases. Further research with larger sample sizes and biochemical analyses is needed to clarify the extent of antioxidant influence on nifedipine-induced reproductive alterations.

## 1 Introduction

Reproductive health is a cornerstone of species survival in both humans and animals. It ensures not only individual fitness but also the perpetuation and propagation of genetic materials across generations (1), making the promotion of reproductive health imperative for sustainable population dynamics and biodiversity conservation (2). However, any disruption in reproductive processes can threaten the existence of species over time (2, 3). Infertility, the inability to conceive after a specified period of mating or unprotected sexual intercourse, is a major impediment to reproductive health in both animals and humans (4–7). Its causes are multifactorial, including nutritional deficiencies, environmental toxins, stress, medical conditions, certain medications, and genetic factors (5, 6, 8–11). Although many etiologies are well characterized, idiopathic infertility remains a diagnostic challenge and continues to limit reproductive success (8).

Some FDA (Food and Drug Administration) approved drugs for certain medical conditions, particularly antihypertension, have been implicated in male reproductive dysfunction due to their adverse effects on spermatogenesis and gonadal structure (12, 13). Nifedipine, a potent FDA approved antihypertensive agent, exerts action by blocking voltage-gated L-type calcium channels in the vascular smooth muscles and myocardial cells during the depolarization phase, leading to reduced intracellular calcium, decreased peripheral resistance, and improved coronary perfusion (14, 15). Despite its therapeutic benefits in hypertension, angina pectoris, and preterm labor, nifedipine has been linked to impaired spermatogenesis, reduced sex hormone levels, oxidative stress, and testicular degeneration (16–19). Excessive generation of reactive oxygen species (ROS) during drug-induced stress can overwhelm the endogenous antioxidant system, resulting in lipid peroxidation, protein modification, DNA damage, and reduced sperm function (20–22).

Antioxidants have been reported to play a crucial role in mitigating the harmful effects of oxidative stress on cells (22, 23). Some natural agents, such as garlic and vitamin C, have potent free radical-scavenging properties, making them useful for mitigating oxidative stress-induced damage (24). Vitamin C, a water-soluble vitamin, is a powerful exogenous antioxidant with the potential to scavenge excessive ROS produced in the system (25, 26). It plays a role in immune function and is involved in the biosynthesis of collagen, carnitine, and certain neurotransmitters (25, 27, 28). Studies have shown that vitamin C can improve sperm count, morphology, and overall reproductive function (29–31). Garlic (*Allium savitum*), on the other hand, contains several sulfur-containing bioactive compounds notably allicin (a lipophilic bioactive compound) and S-allyl cysteine (a hydrophilic compound), which exhibit the antioxidant, anti-inflammatory, and immunomodulatory properties (32). It has also been reported to enhance blood circulation, libido, testosterone production, spermatogenesis, cardiovascular health and testicular function (33–36).

Synergistic effects are expected when vitamin C and *Allium sativum* are administered together. Vitamin C primarily functions in the aqueous cellular compartments, where it neutralizes superoxide radicals, hydroxyl radicals, and peroxynitrate, regenerates other antioxidants, and prevents oxidative DNA damage (25, 26). Conversely, garlic contains both hydrophilic (e.g., S-allyl cysteine, S-allyl mercaptocysteine) and lipophilic organosulphure compounds (e.g., allicin, diallyl disulphide, diallyl trisulphide) (32), enabling it to function across both aqueous and lipid-rich cellular compartments. The lipophilic compounds integrate into biological membranes where they inhibit lipid peroxidation and stabilize mitochondrial structures (20–22), while the hydrophilic compounds neutralize ROS in cytosolic environments. This dual hydrophilic–lipophilic antioxidant capacity of garlic, combined with the strong hydrophilic ROS-scavenging action of vitamin C, provides broader oxidative protection and may yield superior reproductive benefits compared to either agent alone. Given the antioxidant and reproductive-enhancing properties of vitamin C and garlic, this study aimed to evaluate their individual and combined effects on nifedipine-induced reproductive toxicity in adult male albino rats. The findings may provide valuable insights into natural antioxidant-based interventions for managing drug-induced infertility in males.

## 2 Materials and methods

### 2.1 Animal use and care approval

The ethical clearance for this study was granted by the Institutional Animal Care and Use Committee (IACUC) of the University of Nigeria, Nsukka (Approval No. FVM-UNN-IAUCC-2025-02/207) on February 28, 2025. All procedures involving experimental animals were carried out humanely and in strict accordance with the guidelines set by the IACUC and the National Research Council for the care and use of laboratory animals in scientific research (37).

### 2.2 Experimental animals

**Figure 1:**
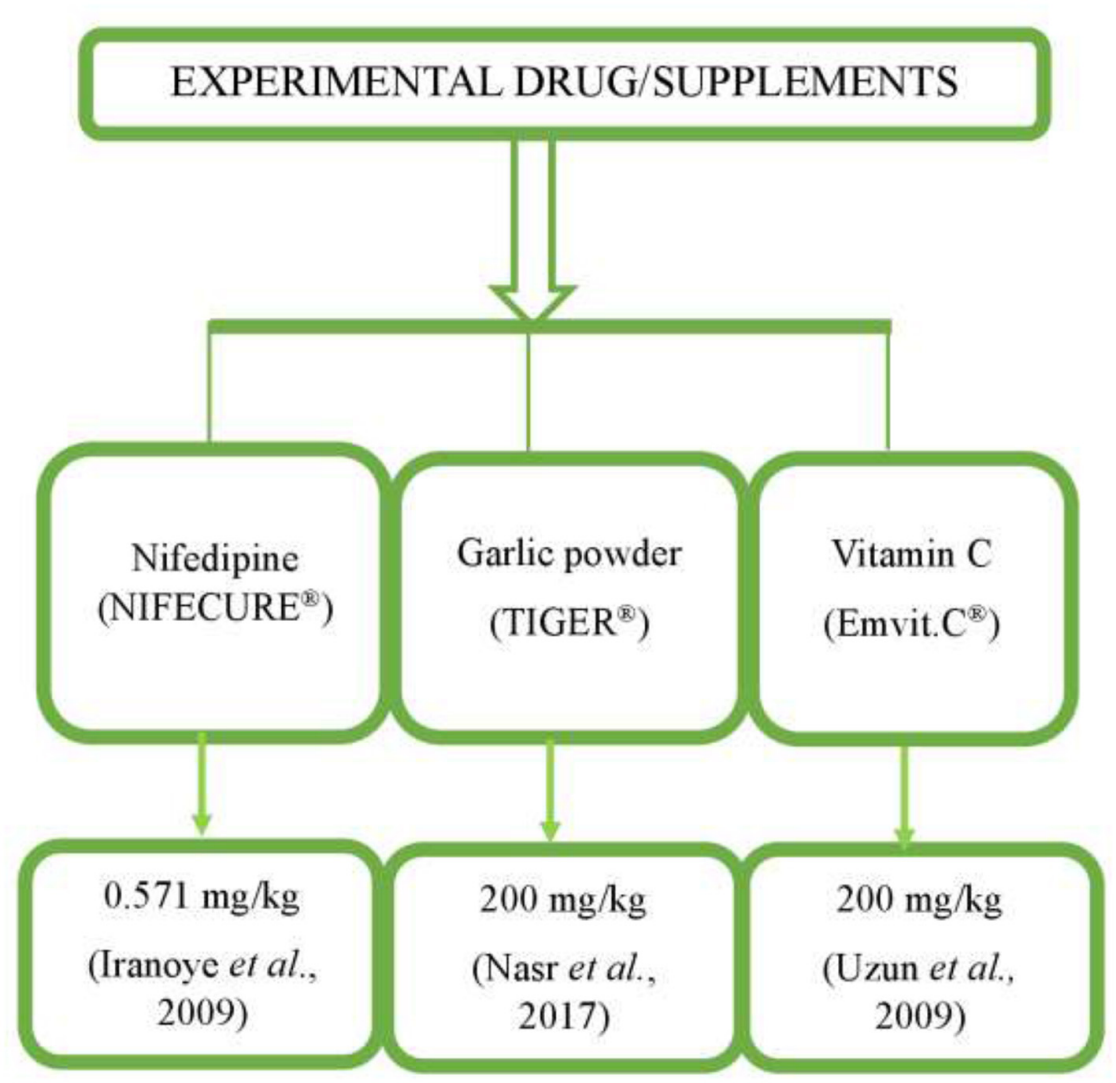
Schematic chart of experimental drug and supplements used.

**Figure 2:**
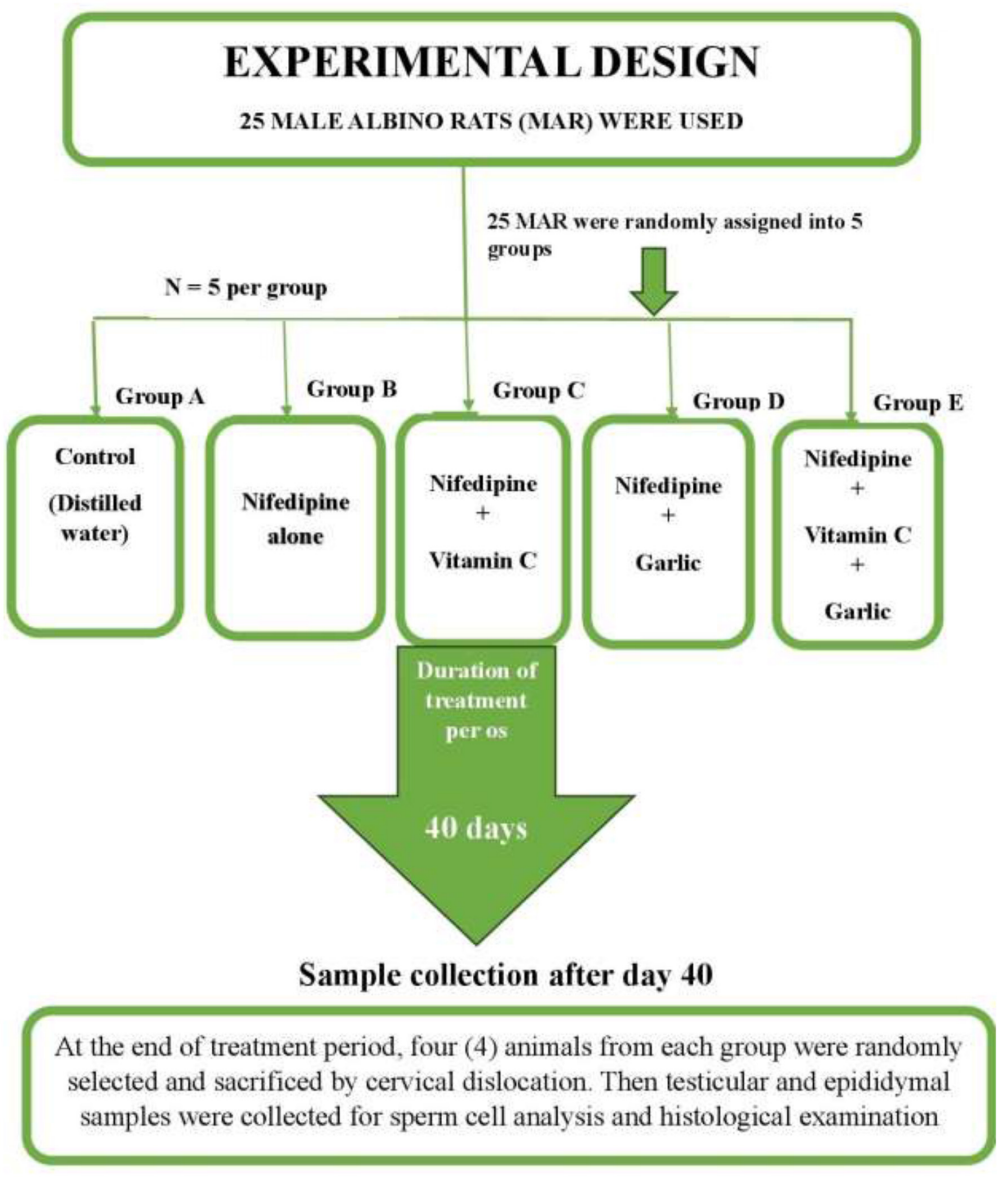
A schematic flowchart of the experimental design.

Twenty-five (25) adult male albino rats (MARS), each weighing between 150 and 250 g, were housed in the laboratory animal section of the Department of Veterinary Obstetrics and Reproductive Diseases, Faculty of Veterinary Medicine, University of Nigeria, Nsukka. The rats were housed in clean, well-ventilated aluminum wire-mesh cages under controlled environmental conditions suitable for subtropical climate, including an ambient room temperature of 25 °C, and maintained under a 12-h light/12-h dark cycle with a relative humidity of 50%−69%. The animals were acclimatized for 3 weeks prior to the start of the research. Throughout the study, they had unlimited access to commercial rat chow and water. Details of the experimental drugs are presented in Tables 1, 2 and Figure 1, while and the experimental study design is presented in Figure 2. The experimental animals were randomly assigned into five groups (*n* = 5 per group) as follows:

- Group A (positive control): received distilled water only.
- Group B (negative control): received nifedipine only.
- Group C: received nifedipine in combination with vitamin C.
- Group D: received nifedipine in combination with garlic powder.
- Group E: received nifedipine in combination with both vitamin C and garlic powder.

**Table 1:** The concentrations and volumes of garlic used.

**Day**	**Garlic used (mg)**	**Volume of solvent (ml)**	**Concentration (mg/ml)**
1–8	420	3.5	120.00
9–18	650	4.5	144.44
19–37	720	4.5	160.00
37–40	600	4.0	150.00

Each rat received an equal volume of the suspension via oral gavage once daily.

**Table 2:** The concentrations and volumes of Vitamin C used.

**Day**	**Vitamin C used (mg)**	**Volume of solvent (ml)**	**Concentration (mg/ml)**
1–8	400	3.5	114.29
9–18	700	5.5	127.27
19–37	800	5.5	145.45
37–40	600	4.5	133.33

The calculated daily volume was administered orally to each rat using a calibrated gavage needle.

Only the nifedipine preparation required dimethyl sulfoxide (DMSO) as a co-solvent to ensure complete dissolution. Vitamin C and garlic were dissolved in distilled water alone. A separate DMSO-only vehicle control group was not included; however, the actual amount of DMSO administered per rat was approximately 5.7 μl, corresponding to 0.03 ml/kg, a level far below reported toxicological thresholds in rodents, which may not produce toxic effects (38). Therefore, any potential solvent-related effects are expected to be negligible. The entire experimental treatment period lasted 40 days, after which all animals were sacrificed, and reproductive samples collected on day 41.

### 2.3 Experimental drugs

#### 2.3.1 Nifedipine

Nifedipine tablet (NIFECURE^®^, 20 mg; manufactured by Unicure Pharmaceutical Limited, India) was used in this study. The dose of nifedipine (0.571 mg/kg body weight) was selected based on previous experimental studies in rats, particularly Iranoye et al. (17), which demonstrated that this dosage produces measurable reproductive and testicular effects. To prepare the stock solution, 10 mg of nifedipine was accurately weighed using a sensitive electronic balance. It was first dissolved in 0.5 ml of dimethyl sulfoxide (DMSO) to ensure complete solubility. This solution was then diluted with 14.1 ml of distilled water to obtain a final volume of 14.6 ml, resulting in a drug concentration of 0.68 mg/ml. The required volume of the drug administered to each animal was calculated using the formula:

Volume to administer (ml) = (Dosage (mg/kg) × Body weight of rat (kg)) ÷ Drug concentration (mg/ml)

Each rat received its dose via oral gavage once daily for the entire duration of the experiment.

#### 2.3.2 Garlic (Allium sativum)

Commercial garlic powder (TIGER^®^, 100% Allium sativum) was used at a dosage of 200 mg/kg body weight, following the protocol of Nasr et al. (39). The working solutions were prepared at different stages of the experiment to account for changes in body weights of the rats. For each dosing period, the total weight of the five rats in the garlic-treated group was recorded, and the required quantity of garlic was calculated as:

Garlic required (mg) = 200 mg/kg × Total body weight of rats (kg).

The garlic powder was dissolved in distilled water to form a uniform suspension. The concentration used and corresponding volumes are shown in Table 1.

#### 2.3.3 . Vitamin C (ascorbic acid)

Vitamin C tablets (Emvit-C^®^, 100 mg; manufactured by Emzor Pharmaceutical Industries Ltd., Nigeria) were used. The dose administered was 200 mg/kg body weight, as described by Uzun et al. (40). At each stage of the study, the total body weight of the five rats in the vitamin C-treated group was used to calculate the amount of vitamin C needed using the formula:

Vitamin C required (mg) = 200 mg/kg × Total body weight of rats (kg).

The vitamin C tablets were crushed and dissolved in distilled water. The working solutions were prepared freshly as needed, and the concentrations used are shown in Table 2.

### 2.4 Sperm analysis

#### 2.4.1 Sperm motility

Sperm motility (%) was assessed using the sperm diffusion method previously reported by Klinefelter et al. (41). The epididymis was carefully dissected from the testis, and the cauda epididymis was separated from the vas deferens. The cut end of the tubule was immersed in a drop of pre-warmed phosphate-buffered saline (PBS) with pH and temperature of 7.4 and of 37 °C, respectively on a clean microscope slide to facilitate sperm dispersion. After 2 min, the tissue was removed, and the sample was incubated for 5 min until sperm cells were adequately dispersed. Motility evaluation was performed under a phase-contrast microscope (Motic B3; Motic, Carlsbad, CA, USA) at × 100 magnification, using a stage slide warmer set at 37 °C (TCS-100; Amscope, Irvine, CA, USA). A total of 200 spermatozoa were counted per sample, and motility was expressed as the percentage of motile sperm relative to the total counted.

#### 2.4.2 Sperm viability

Evaluation of the sperm viability was determined using the eosin–nigrosin vital staining technique. Equal volumes (50 μl each) of cauda epididymal sperm suspension and eosin–nigrosin stain were mixed for 30 s. A thin smear was prepared on a clean slide and air-dried. Live sperm (with unstained inner outline) and dead sperm (with pink-stained inner outline) were identified under light microscopy (Motic B3; Motic, Carlsbad, CA, USA) at × 400 and × 1,000 magnification under oil immersion. Two hundred sperm cells were counted, and viability was expressed as the percentage of live sperm. Micrographs were captured using the Moticam 2.0 imaging system (Motic, Carlsbad, CA, USA) (42).

#### 2.4.3 Epididymal sperm concentration

The cauda epididymal tissue was minced and homogenized in 2 ml of PBS (pH 7.4). A 1:200 dilution of the homogenate was prepared using sperm dilution fluid containing formalin and gentian violet stain. Sperm counts were performed with a haemocytometer (Weber, England), and results were expressed as the number of spermatozoa per milliliter or per gram of cauda epididymis (42).

#### 2.4.4 Epididymal sperm morphology

Sperm morphology was evaluated from wet mounts of cauda epididymal sperm suspension at × 400 and × 1,000 magnification using phase-contrast microscopy (Motic B3; Motic, Carlsbad, CA, USA). Additional morphology assessment was conducted using the eosin–nigrosin staining method. Two hundred sperm cells were examined, and abnormalities were classified and recorded as the percentage of normal sperm, total sperm abnormalities (TSA), and specific abnormality types (42).

#### 2.4.5 Testicular sperm reserve and concentration

The tunica albuginea was carefully removed from the testis, and a known weight of the parenchyma was thoroughly minced using sterile surgical scissors. The minced tissue was homogenized in 2 ml of phosphate-buffered saline (PBS, pH 7.4) to release the sperm cells. A 1:200 dilution of the homogenate was prepared in sperm dilution fluid containing formalin as a fixative and gentian violet as a staining agent to enhance visibility of spermatozoa. Elongated spermatids and spermatozoa were counted using a haemocytometer (Weber, England). Testicular sperm reserve (TSR) was determined by multiplying the testicular sperm concentration by the total weight of the testis. TSR is expressed as the number of sperm cells per gram testis (42).

### 2.5 Histology of the testes

Histological evaluation of the testes and the epididymis was performed using a previously described procedure by Slaoui and Fiette (43). The tissues were carefully harvested and immediately fixed in Bouin's solution for 24 h to preserve cellular and structural integrity. Following fixation, the samples were dehydrated through a graded series of ethanol, cleared in xylene, and embedded in paraffin wax blocks. Tissue sections of 5 μm thickness were obtained using a rotary microtome blade and mounted on clean glass slides. The sections were then stained with hematoxylin and eosin (H&E) to differentiate nuclear and cytoplasmic components before being examined under a light microscope for histopathological evaluation.

### 2.6 . Organ weights and somatic index

Randomly selected MARs were euthanized by cervical dislocation (44) to harvest the paired testes and epididymis for organosomatic index (OSI) assessment. The weight of the organs was measured using an electronic weighing scale (Ohaus, Pine Brook, NJ, USA). Testis length and width were measured using a vernier caliper. The organosomatic index (OSI, %) was calculated by dividing the organ weight (g) by the body weight (g) and multiplying by 100 as previously reported by Oliveira et al. (45).

### 2.7 Data analysis

Data were analyzed using SPSS version 21 (IBM Corp., USA). Normality of all continuous variables was assessed using the Shapiro–Wilk test. Homogeneity of variance across treatment groups was evaluated using Levene's test. Most parameters, including sperm motility, viability, epididymal sperm concentration (ESC), testicular sperm concentration (TSC), and sperm reserves (ESR, TSR), satisfied normality assumptions (Shapiro–Wilk *P*-values ranging from 0.054 to 0.98). Levene's test indicated that the majority of variables met the homogeneity of variance requirement (*P*-values ranging from 0.087 to 0.700).

Mild deviations from normality were observed in a few cases (e.g., A, D and E groups for ESC, motility, and viability, respectively), and unequal variances was detected for GSI, ESR, TSR, and motility (Levene's *P* < 0.05). However, given the small and equal group sizes (*n* = 4 per group) and ANOVA's robustness to minor assumption violations, the data were analyzed using one-way ANOVA followed by Fisher's Least Significant Difference (LSD) *post-hoc* test. Statistical significance was established at *P* ≤ 0.05. Results in tables are presented as mean ± standard error of the mean (SEM), while error bars in figures represent significant differences between groups.

## 3 Results

### 3.1 Sperm analysis of male albino rats (MAR)

The mean values of sperm motility and viability in male albino rats treated with vitamin C, garlic and their combination following nifedipine administration are presented in Table 3. Mean epididymal and testicular sperm concentrations and reserves are presented in Table 4.

**Table 3:** Mean sperm motility and viability in male albino rats administered nifedipine and treated with vitamin C, garlic, or their combination.

**Parameters (units)**	**Groups**				
	**Control**	**Nifedipine alone**	**Nifedipine** + **vitamin C**	**Nifedipine** + **garlic**	**Nifedipine** + **vitamin C** + **garlic**
Motility (%)	83.25 ± 1.65^a^	38.90 ± 2.53^b^	41.25 ± 6.95^b^	35.52 ± 5.32^b^	45.00 ± 7.5^b^
Viability (%)	94.00 ± 0.98^a^	88.03 ± 1.87^b^	90.28 ± 1.21^a, b^	89.03 ± 1.45^b, c^	91.18 ± 1.29^a, b^

Means ± SEM (standard error of the mean) within the same row bearing different superscript letters (a, b, c) indicate significant differences at *p* ≤ 0.05, whereas means with Superscripts such as ‘ab' indicate values that do not differ from groups labeled with either ‘a' or ‘b' (*P* > 0.05).

**Table 4:** Mean epididymal and testicular sperm concentrations and reserves of male albino rats treated with Vit. C, garlic, and and a vitamin C + garlic combination following nifedipine administration.

**Parameters (units)**	**Groups**				
	**Control**	**Nifedipine alone**	**Nifedipine** + **vitamin C**	**Nifedipine** + **garlic**	**Nifedipine** + **vitamin C** + **garlic**
ESC (sperm/ml)	2.55 × 10^7^ ± 2.34 × 10^6a^	1.80 × 10^7^ ± 1.06 × 10^6b^	1.91 × 10^7^ ± 1.70 × 10^6b^	2.22 × 10^7^ ± 1.11 × 10^6c^	2.26 × 10^7^ ± 2.12 × 10^6c^
ESR (sperm/g epididymis)	3.10 × 10^8^ ± 1.91 × 10^7a^	2.02 × 10^8^ ± 7.20 × 10^6a^	2.16 × 10^8^ ± 2.13 × 10^7a^	2.34 × 10^8^ ± 9.87 × 10^6a^	2.41 × 10^8^ ± 1.46 × 10^7a^
TSC (sperm/ml)	9.66 × 10^6^ ± 8.97 × 10^5a^	7.38 × 10^6^ ± 1.20 × 10^5b^	8.26 × 10^6^ ± 9.15 × 10^5c^	9.08 × 10^6^ ± 1.14 × 10^6a, c^	9.69 × 10^6^ ± 1.30 × 10^6a^
TSR (sperm/g testis)	1.51 × 10^7^ ± 1.14 × 10^6a^	1.07 × 10^7^ ± 8.74 × 10^5a^	1.11 × 10^7^ ± 7.99 × 10^5a^	1.20 × 10^7^ ± 1.32 × 10^6a^	1.37 × 10^7^ ± 1.29 × 10^7a^

Means ± SEM with different superscripts (a, b, c) in the same row differ significantly (*P* ≤ 0.05); identical superscripts such as ‘ab' indicate values that are not significantly different from groups labeled with either ‘a' or ‘b' (*P* > 0.05).

ESC, epididymal sperm concentration; ESR, epididymal sperm reserve; TSC, testicular sperm concentration; TSR, testicular sperm reserve.

#### 3.1.1 Effects of vitamin C (Vit. C) and garlic on nifedipine-induced abnormal sperm motility in male albino rats

The ameliorative effects of Vit. C and garlic on nifedipine-induced abnormal sperm motility reduction in male albino rats are presented in Table 3. There was a significant decrease in the motility of rat sperm cells in group B (nifedipine) compared to the control (group A) at *P* < 0.001. The mean sperm motility of groups C (Vit. C), D (garlic) and E (Vit. C + garlic) compared to group B (nifedipine) were not statistically significant at *P* = 0.76, 0.661, and 0.432, respectively; however, there was a slight increase in the sperm motility of groups C (Vit. C) and E (Vit. C + garlic) (2.35 and 6.1 % increases, respectively).

#### 3.1.2 Effects of Vit. C and garlic on nifedipine-induced decrease in sperm viability

The ameliorative effects of Vit. C and garlic on nifedipine-induced decrease in sperm viability are presented in Table 3. Statistical analysis showed a significant decrease in the viability of rat sperm cells in group B (nifedipine) compared to the control (group A) at *P* = 0.008. The mean sperm viability of Vit. C, garlic and a combination of the Vit. C and garlic groups compared to the nifedipine group were not statistically significant (*P* > 0.05); however, there was a slight increase in the sperm viability of Vit. C and combination of Vit. C and garlic groups (2.25 and 3.15 % increase, respectively), which were not statistically different from the control group at *P* = 0.167.

#### 3.1.3 . Effects of Vit. C and garlic on nifedipine-induced decrease in epididymal and testicular sperm concentrations in male albino rats (MAR)

The results of the ameliorative effects of Vit. C and garlic on nifedipine-induced decrease in mean epididymal sperm concentration (ESC) and testicular sperm concentration (TSC) are presented in Table 4. Statistical analysis showed a significant decrease in both ESC and TSC in the nifedipine group compared to the control at *P* < 0.001 and 0.001, respectively.

The effect of Vit. C on nifedipine-induced decrease in ESC, was not statistically significant (*P* = 0.097); however, garlic and a combination of the Vit. C and garlic treatment showed significant increases in ESC at *P* < 0.001 (0.42 % increase) and < 0.001 (0.46 % increase), respectively, compared to the nifedipine group.

On the other hand, all supplement-treated groups showed significant protective effects against nifedipine-induced decrease in TSC, i.e. there were significant increases in the TSC of Vit. C, garlic and a combination of the Vit. C and garlic groups compared to nifedipine group at *P* = 0.03, 0.001, and *P* < 0.001, respectively. The percentage increase of these groups was 2.3, 1.7, and 0.88 % respectively.

#### 3.1.4 . Effects of Vit. C and garlic on nifedipine-induced decrease in epididymal and testicular sperm reserves in male albino rats

The results on the protective effects of Vit. C and garlic on nifedipine-induced decreases in mean ESR, and TSR are presented in Table 4. Statistical analysis revealed no significant differences in the mean ESR and TSR of male albino rats in the nifedipine group compared to the control (*P* = 0.130, 0.236, respectively). Furthermore, even though there were slight increases in the mean ESR and TSR in groups C (Vit. C), D (garlic), and E (Vit. C + garlic) compared to group B (nifedipine group), the increases were not significant.

### 3.2 . Epididymal sperm abnormalities of male albino rats (MARs)

#### 3.2.1 . Protective effect of Vit. C and garlic on nifedipine-induced decrease in normal sperm cells in male albino rats

**Figure 3:**
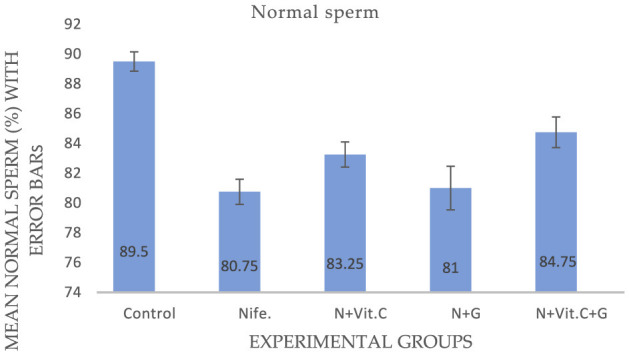
Mean normal sperm cell in male albino rats dosed with Vit. C, garlic and Vit. C/garlic. Data are shown as mean ± SEM (*n* = 4 per group). Nife, nifedipine; N, nifedipine; G, garlic.

The research outcome on the effects of Vit. C and garlic on nifedipine-induced decrease in normal sperm cells are presented in Figure 3. The control group had the highest percentage of normal sperm cells (89.50%), confirming baseline reproductive health. Statistical analysis showed a significant decrease (*P* < 0.05) in the mean normal sperm cells of the nifedipine (group B), nifedipine/vitamin C (group C), nifedipine/garlic (group D) and nifedipine + vitamin C + garlic (group E) groups compared to the control (group A) at *P* < 0.001, *P* = 0.002, *P* < 0.001 and *P* = 0.0011, respectively. Only the nifedipine + vitamin C +garlic group (group E) had a significant 4% ameliorative effect (*P* = 0.027) on nifedipine-induced reproductive disorders. However, groups C and D had nosignificant (*P* > 0.05) ameliorative effect, despite their slight 2.5 and 0.25% increase, respectively.

#### 3.2.2 . Effect of Vit. C and garlic on nifedipine-induced increase in detached head abnormalities of sperm cells in male albino rat (MAR)

**Figure 4:**
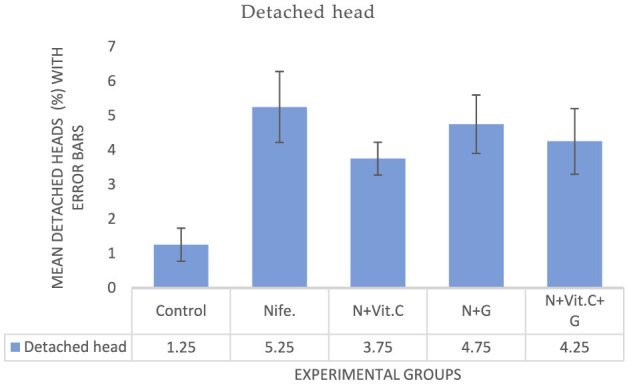
Mean detached head abnormalities of sperm cells in male albino rats (MAR) treated with Vit. C, garlic and Vit. C + garlic. Data are expressed as mean ± SEM (*n* = 4 per group). Nife, nifedipine; N, nifedipine; G, garlic.

The experimental results on the effects of Vit. C and garlic on nifedipine-induced increase in detached head abnormalities of Sperm cells in MARs are presented in Figure 4. Statistical analysis showed a significant increase in the mean percentage of detached heads in the nifedipine group compared to the control (*P* < 0.001). Only the Vit. C group exhibited a mild trend toward improvement, with 1.5% decrease in detached heads compared to the nifedipine group. However, this effect was marginally non-significant (*P* = 0.054).

#### 3.2.3 . Protective effect of Vit. C and garlic on nifedipine-induced increase in fractured neck and coiled tail of sperm cells in male albino rats (MAR)

**Figure 5:**
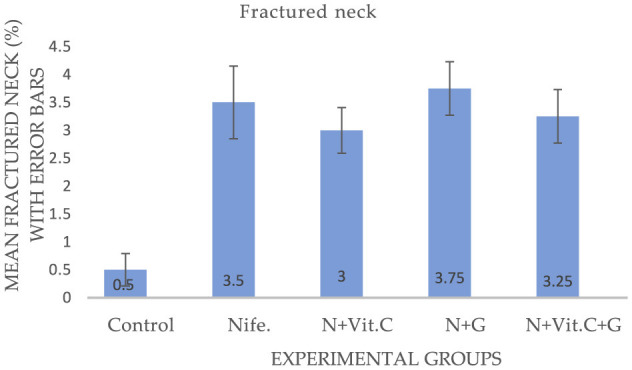
Mean fractured neck of sperm cells in male albino rats (MAR) treated with Vit. C, garlic and Vit. C + garlic. Data are expressed as mean ± SEM (*n* = 4 per group). Nife, nifedipine; N, nifedipine; G, garlic.

**Figure 6:**
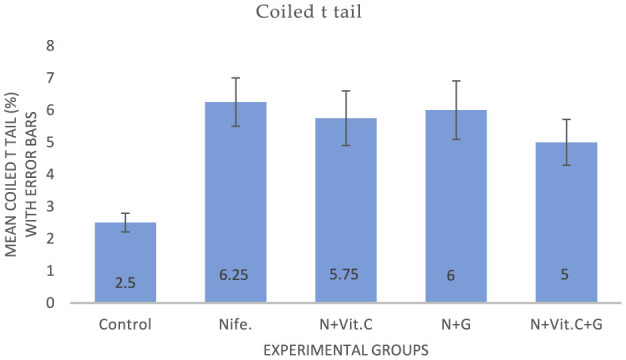
Mean coiled tail of sperm cells in male albino rats (MARs) treated with Vit. C, garlic and Vit. C/garlic. Data are expressed as mean ± SEM (*n* = 4 per group). Nife, nifedipine; N, nifedipine; G, garlic.

The experimental results on the effects of Vit. C and garlic on nifedipine-induced increase in fractured neck and coiled tail abnormalities of Sperm cells in MAR are presented in Figures 5, 6 respectively. Statistical analysis showed a significant increase in the mean fractured neck and coiled tail of sperm cells in MAR in group B (nifedipine) compared to the control (group A) at *P* < 0.001 and *P* = 0.002, respectively. No ameliorative effect was recorded at *P* > 0.05.

#### 3.2.4 . Effect of vitamin C (Vit. C) and garlic on nifedipine-induced bent tail and distal cytoplasmic droplet abnormalities of sperm cells in male albino rats (MARs)

**Figure 7:**
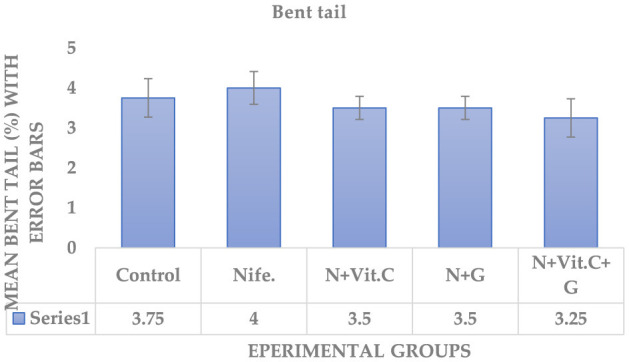
Mean bent tail of sperm cells in MAR dosed with Vit. C, garlic and Vit. C/garlic. Data are shown as mean ± SEM (*n* = 4 per group). Nife, nifedipine; N, nifedipine; G, garlic.

**Figure 8:**
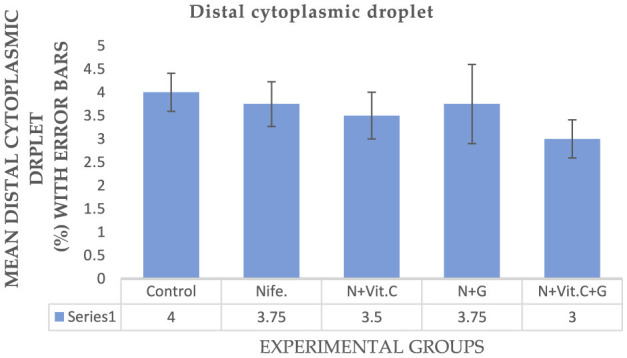
Mean Distal cytoplasmic droplet of sperm cells in male albino rats (MAR) treated with Vit. C, garlic and Vit. C/garlic. Data are expressed as mean ± SEM (*n* = 4 per group). Nife, nifedipine; N, nifedipine; G, garlic.

The experimental results on the protective effects of Vit. C and garlic on nifedipine-induced bent tail and distal cytoplasmic droplet of sperm cells in MARs are presented in Figures 7, 8 respectively. Statistical analysis showed no significant difference (*P* > 0.05) in the mean percentage of bent tail and distal cytoplasmic droplet of MAR sperm cells in group B (nifedipine) compared to the control (group A) and other treatment groups (C, D, and E).

### 3.3 . Histopathological evaluation of testes and epididymides in experimental rat

**Figure 9:**
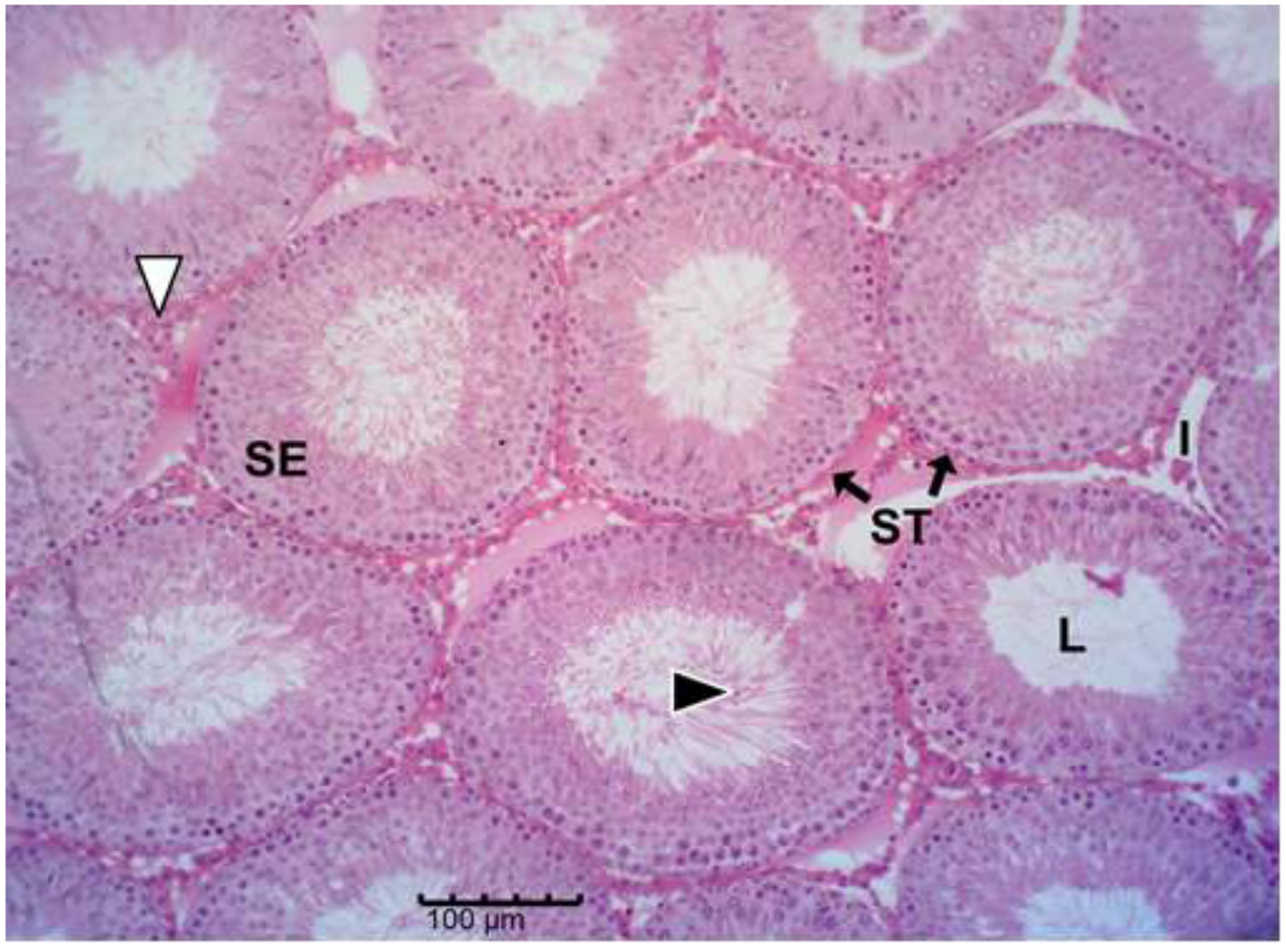
Group A (Control). The micrograph shows normal testicular architecture. Normal seminiferous tubules (ST) with intact stratified seminiferous epithelium (SE), abundant spermatozoa in lumen (L) (black arrow head), and normal interstitial Leydig cells (I).

**Figure 10:**
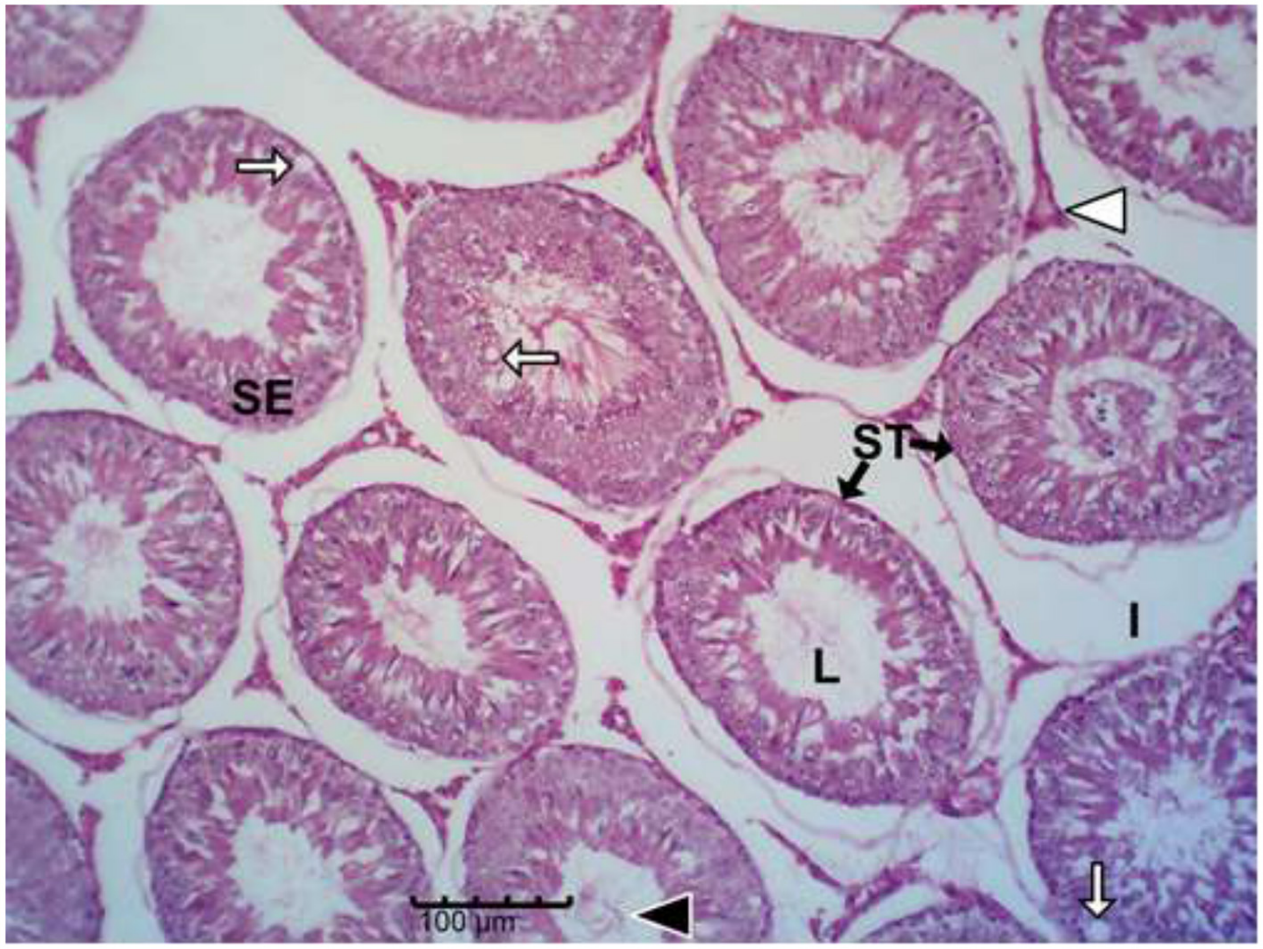
Group B (Nifedipine alone). Marked seminiferous tubular (SE) degeneration with reduced epithelial thickness, germ-cell loss, prominent vacuolation (white arrows), reduced tubular diameter, widened interstitial spaces (I) and scanty presence of spermatozoa (black arrow head) scanty presence of spermatozoa (black arrow head).

**Figure 11:**
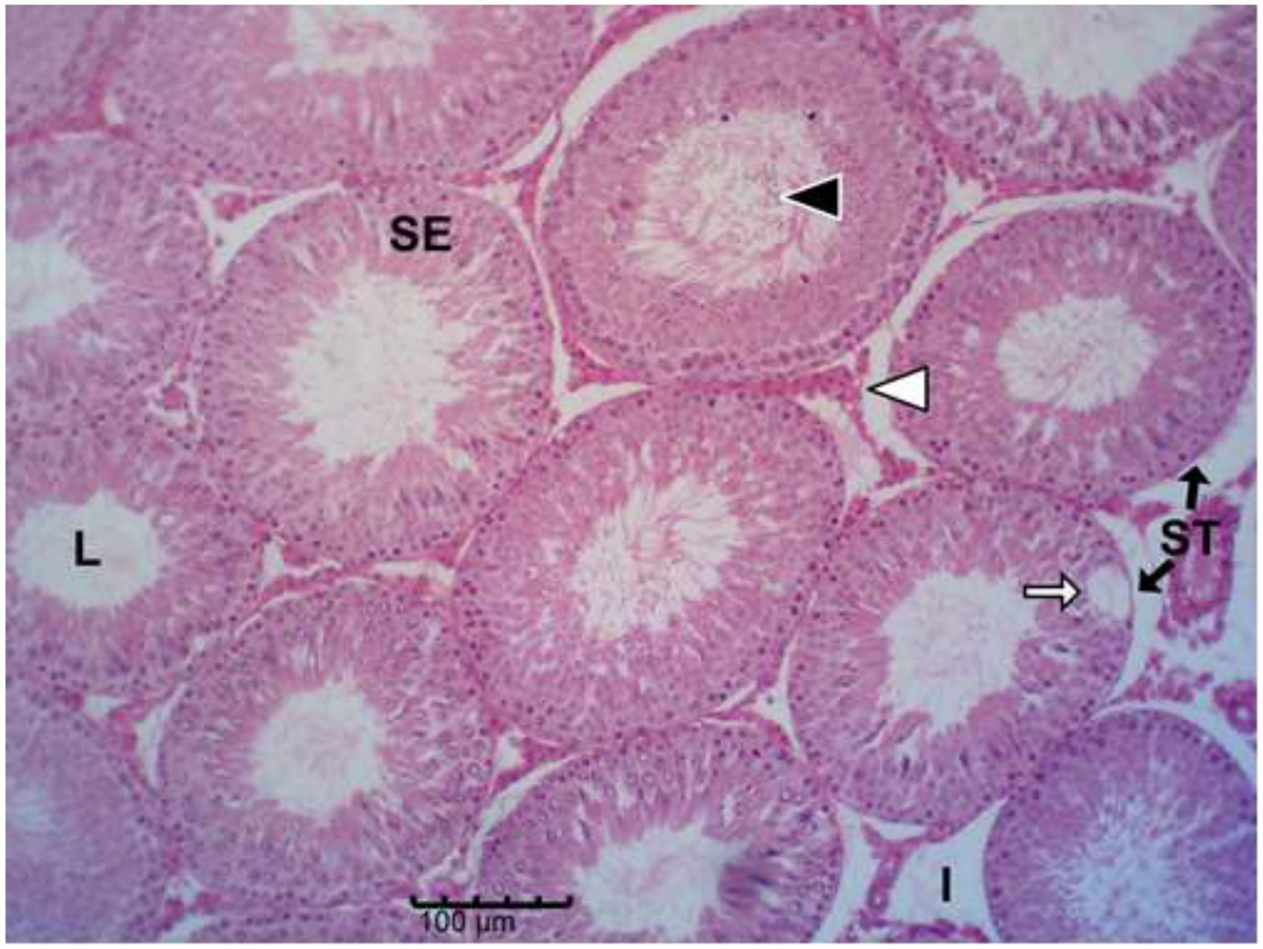
Group C (Nifedipine + Vit. C). Mild tubular degeneration with focal germ-cell loss, mild vacuolation (white arrows) in the seminiferous epithelium (SE) and mild increase in interstitial spaces **(I)**; moderate spermatozoa (white arrows) present in lumen (L).

**Figure 12:**
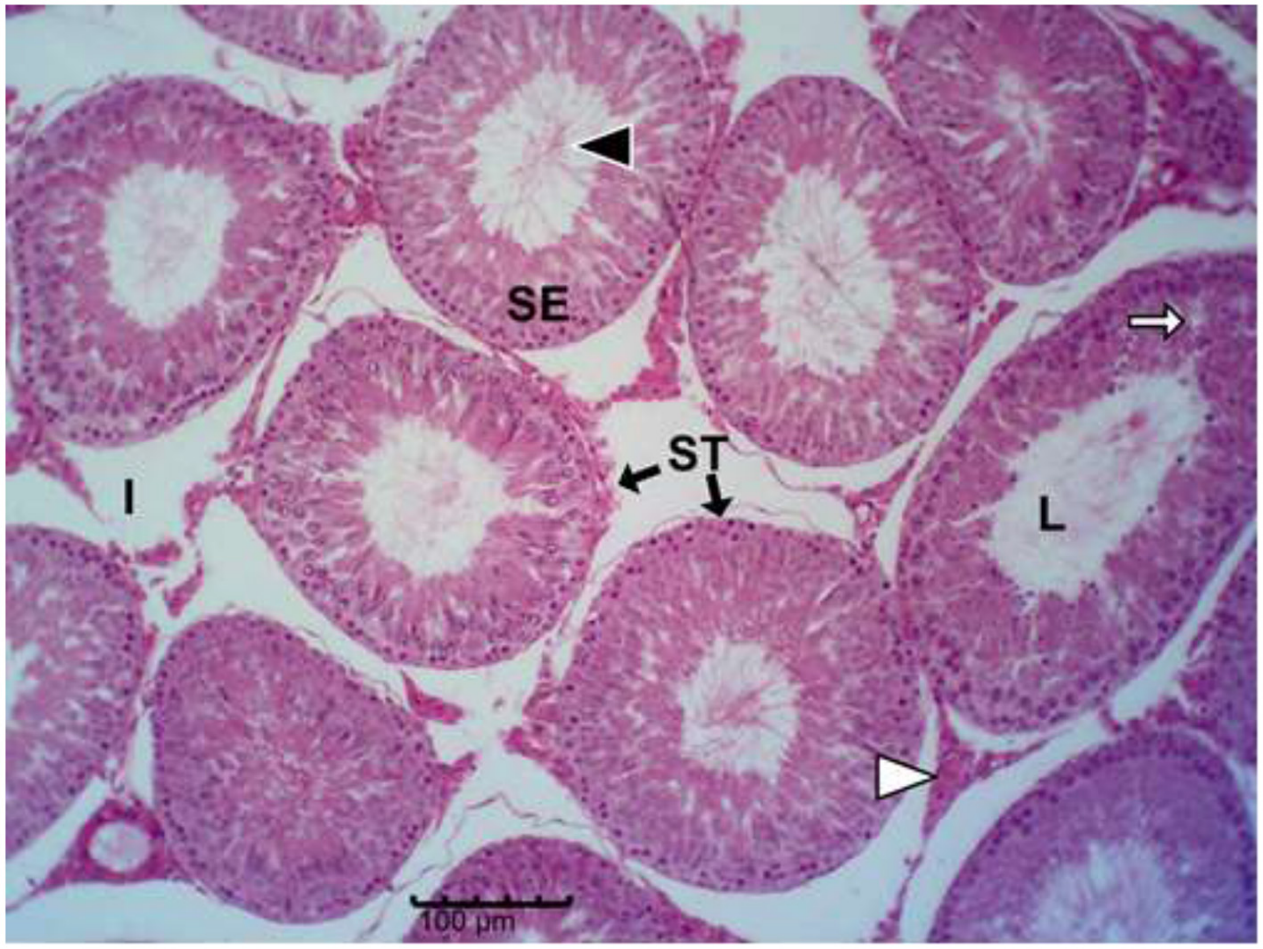
Group D (NIfedipine + Garlic). Moderate tubular degeneration, germ-cell depletion, marked increase in interstitial spaces (I) and noticeable epithelial vacuolation (white arrows) in the SE; moderate spermatozoa (black arrow head) in lumen.

**Figure 13:**
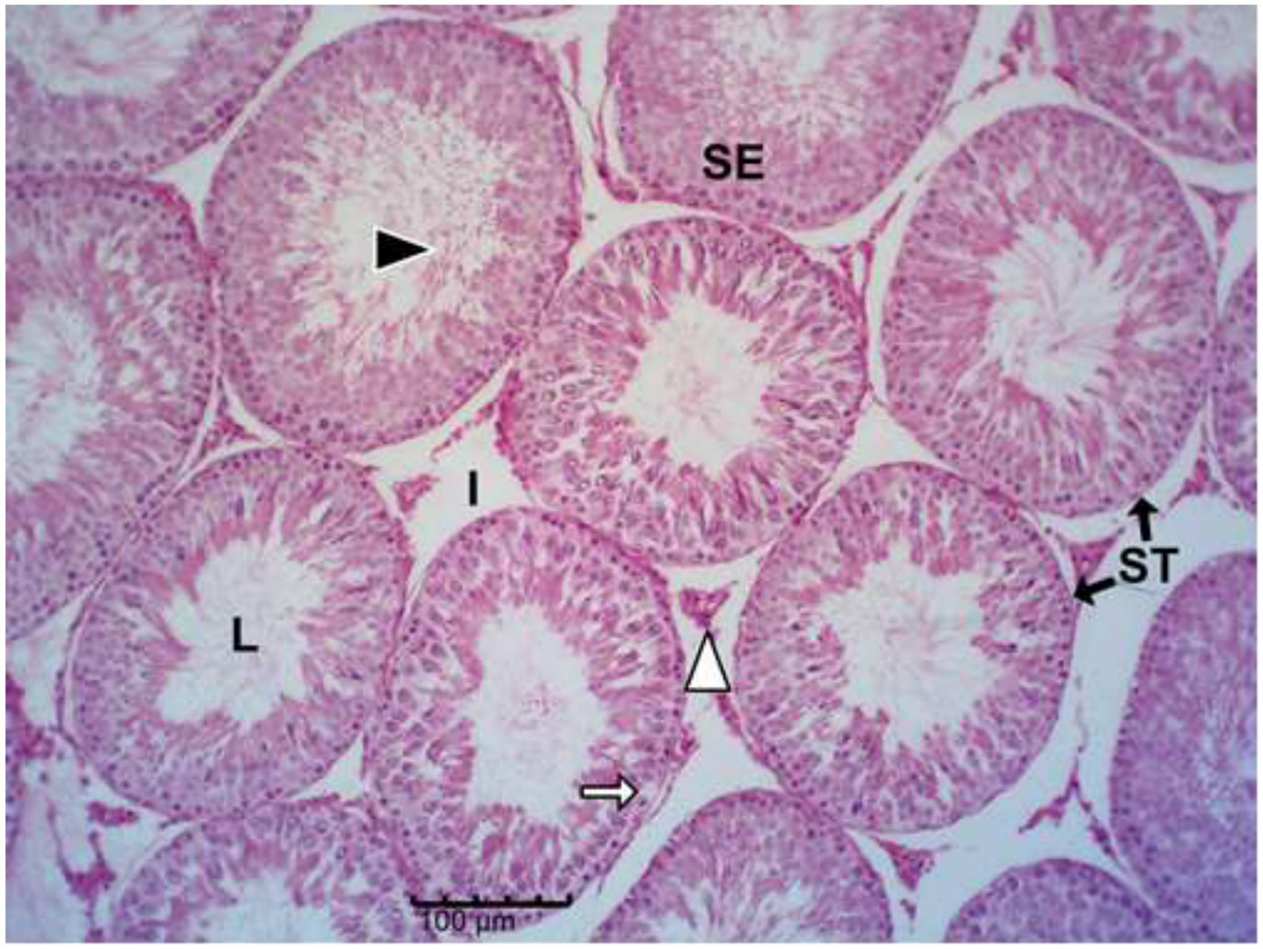
Group E (Nifedipine + Vit. C + Garlic). Moderate degeneration with germ-cell loss, vacuolation (white arrows), and expanded interstitial spaces (I); moderate luminal spermatozoa (black arrow head).

**Figure 14:**
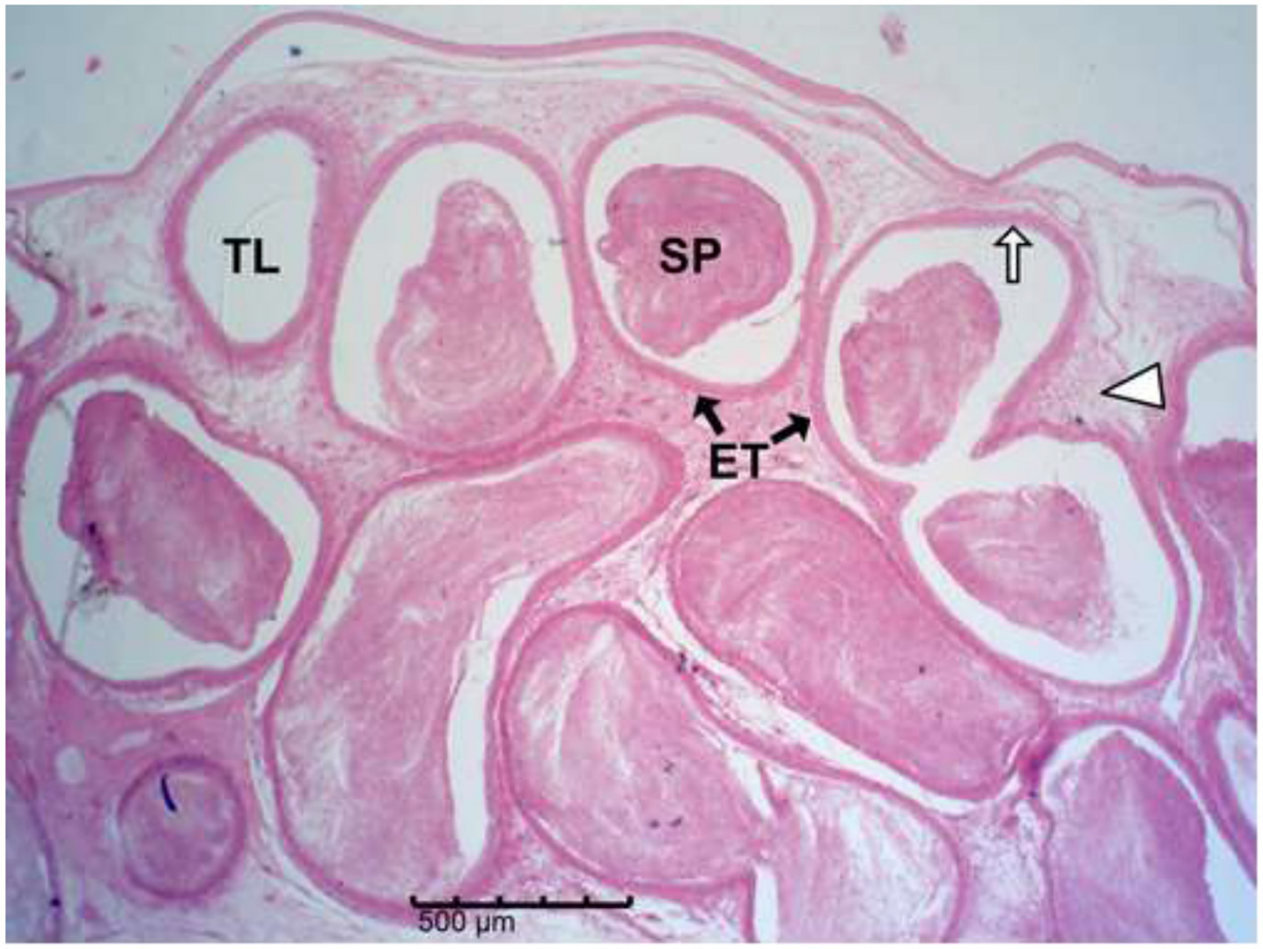
Group A (Control). Normal epididymal tubules (ET) with intact epithelial lining (white arrow), normal interstitial connective tissue (white arrow head), and abundant luminal spermatozoa (SP).

**Figure 15:**
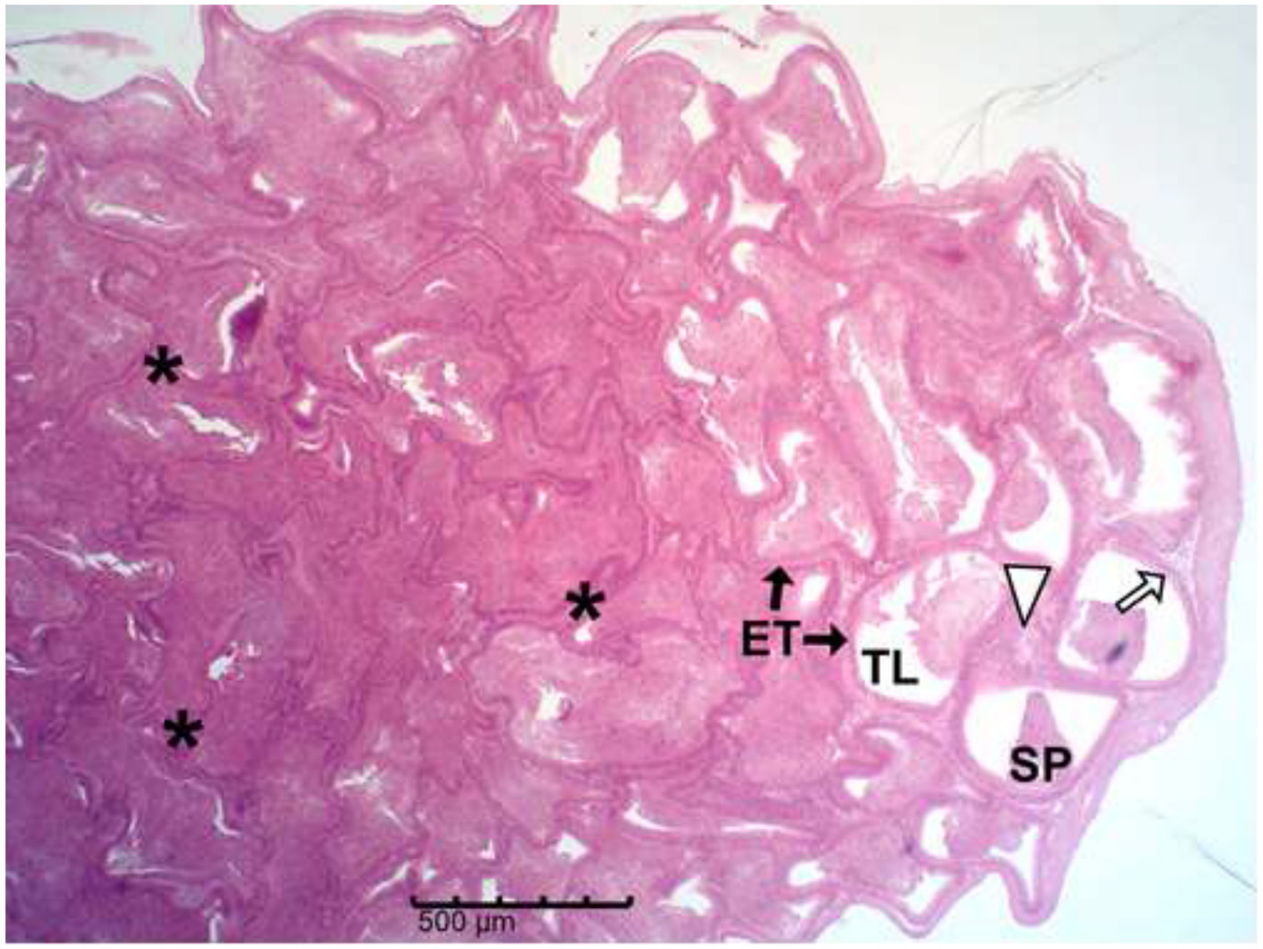
Group B (Nifedipine). Micrograph shows evidence severe epididymal tubular (ET) degeneration and distortion, intertubular connective tissue (white arrow head) and epithelial cell layer loss, basement-membrane thickening, fibrosis (*), and markedly reduced luminal spermatozoa (SP) in the Tubular lumen (TL).

**Figure 16:**
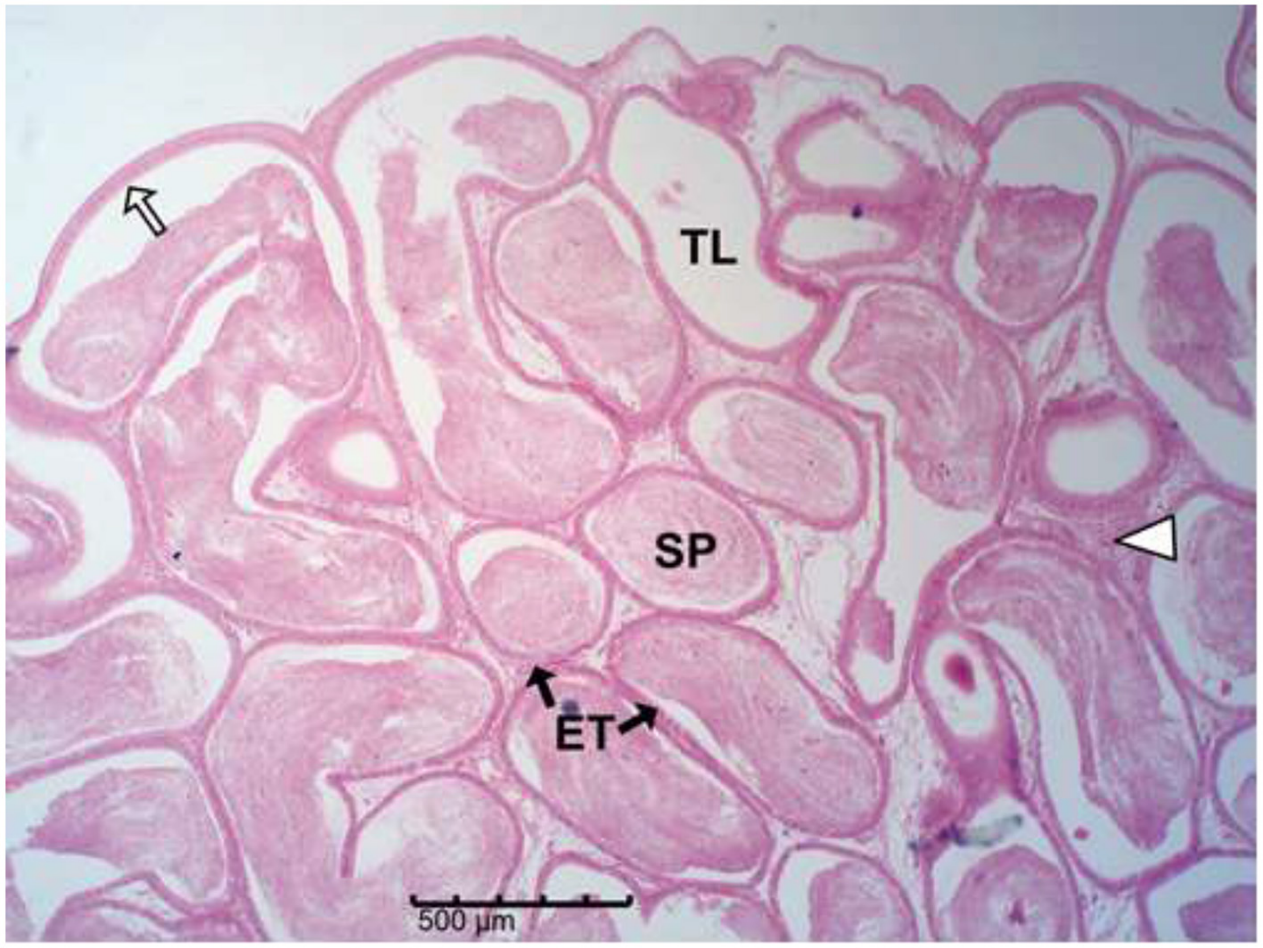
Group C (Nifedipine + Vit. C). Mild degeneration with slightly reduced tubular diameter and loss of intertubular connective tissue (white arrow head); tubules largely preserved with good epithelial integrity (white arrow) and high luminal sperm density (SP).

**Figure 17:**
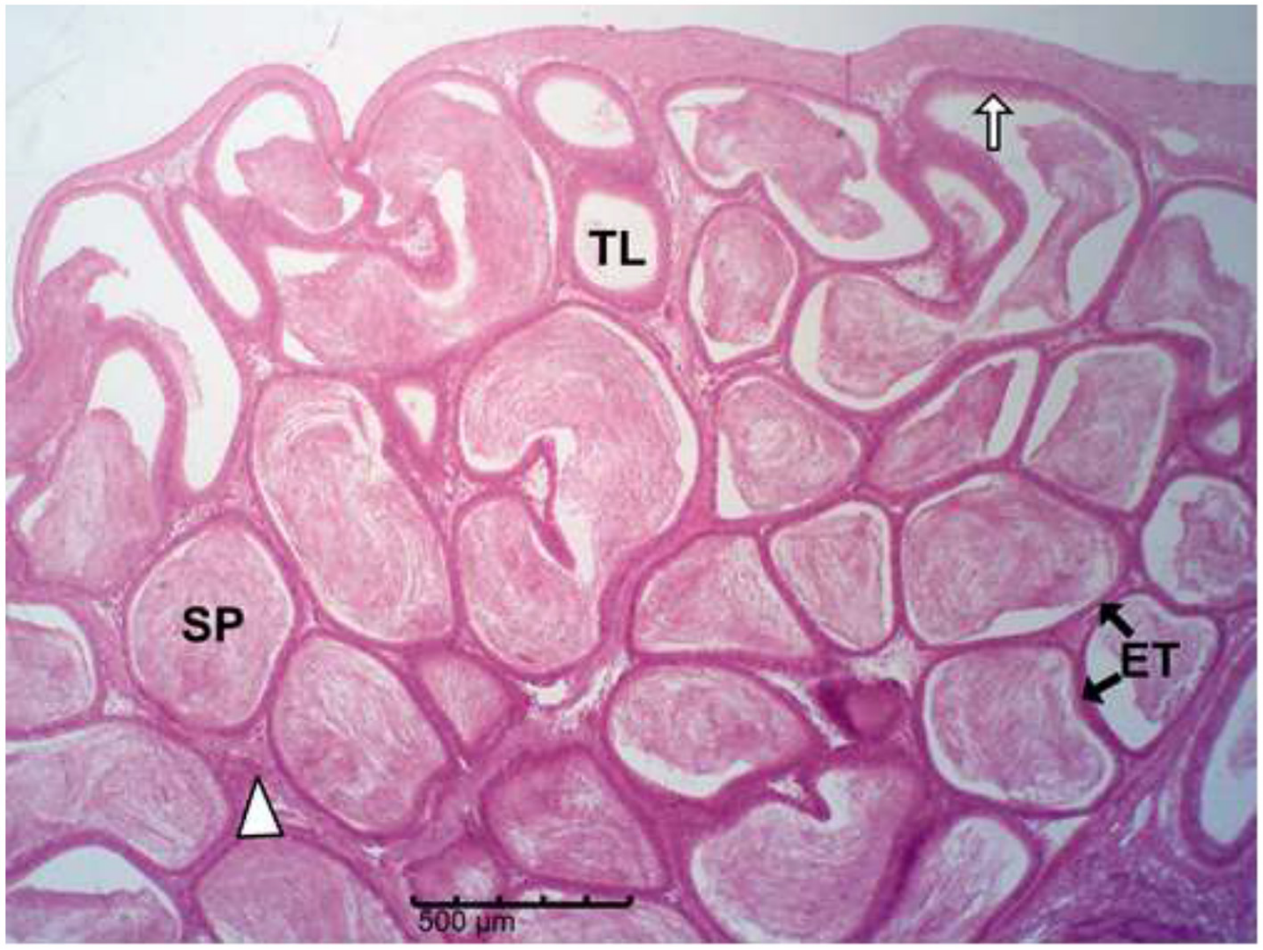
Group D (Nifedipine + Garlic). Micrograph shows mild degeneration, reduced tubular diameter and loss of intertubular connective tissue (white arrow head); luminal spermatozoa remain relatively abundant (SP).

**Figure 18:**
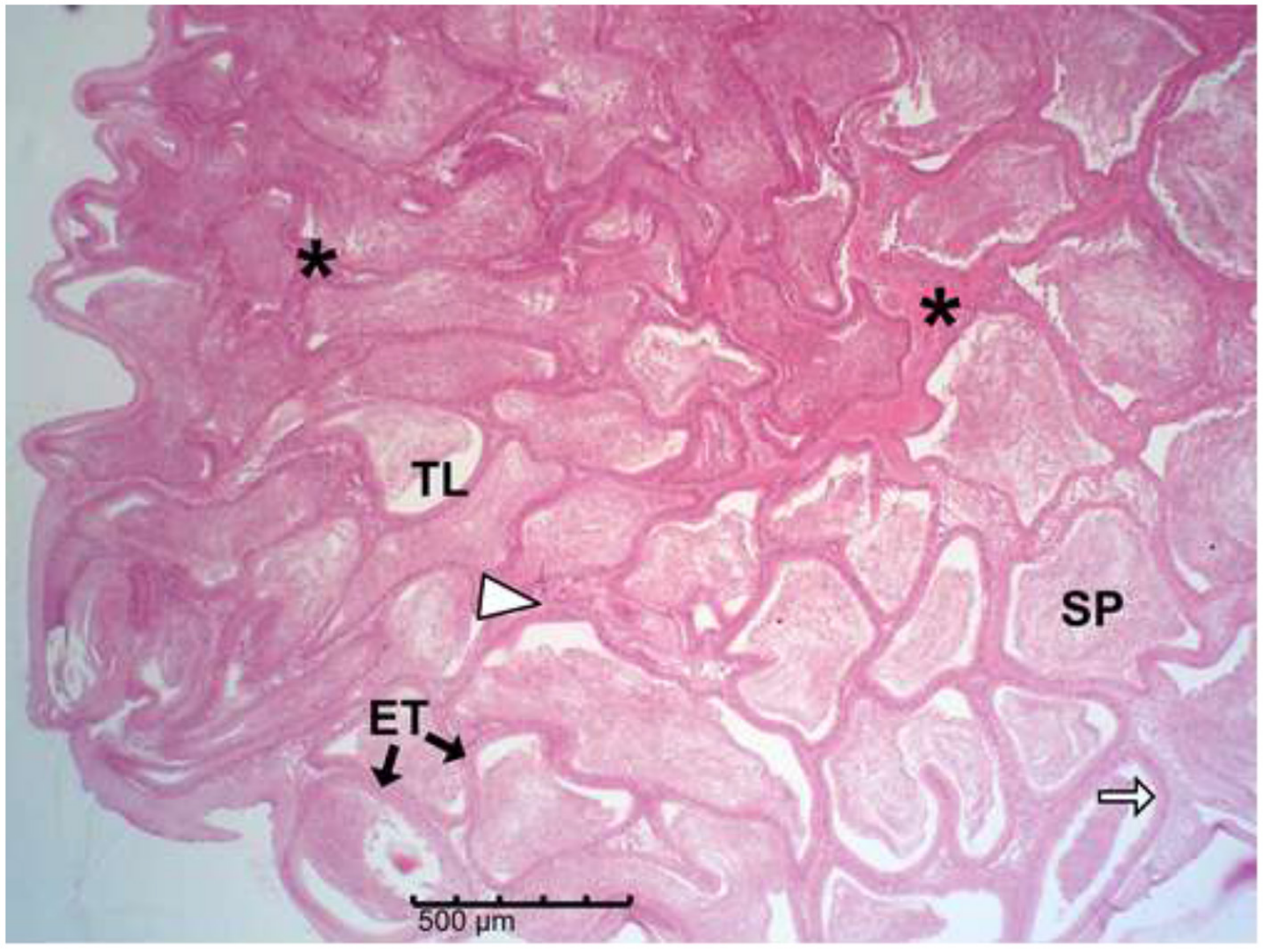
Group E (Drug + Vit. C + Garlic). Localized moderate-to-severe degeneration with epithelial loss and intertubular connective tissue (white arrow head), tubular distortion, fibrosis (*), and moderate luminal spermatozoa (SP).

The histology results of the experimental rat testes and epididymides are presented in Figures 9–18. Marked degeneration of both the testes and the epididymides were recorded in the nifedipine group compared to the control group (Figures 9, 10, respectively). Mild improvement on nifedipine-induced reproductive disorders in rat testes and epididymides were recorded as shown in Figures 11–13, 16–18, respectively.

Histopathology of rat testes across treatment groups (H&E; scale bar = 100 μm; Figures 9–13).

Histopathology of rat cauda epididymides across treatment groups (H&E; scale bar = 500 μm; Figures 14–18).

### 3.4 . Effect of nifedipine on mean body and relative organ weights and the protective effects of vitamin C and garlic

The results on effect of nifedipine on mean body and relative organ weights and the protective effects of vitamin C and garlic are presented in Table 5. The ANOVA results showed significant differences in the mean rat body weight and testicular weight at *P* = 0.034 and 0.009, respectively. No significant differences were found in gonadosomatic index (GSI), epididymal weight, tail weight and epididymosomatic index (ESI) at *P* = 0.416, 0.246, 0.68 and 0.413 respectively.

**Table 5:** The Mean body weights and relative organ weights of male albino rats induced with nifedipine and treated with garlic and vitamin C.

**Parameters**	**Group**				
	**Control**	**Nifedipine alone**	**Nifedipine** + **vitamin C**	**Nifedipine** + **garlic**	**Nifedipine** + **vitamin C** + **garlic**
Rat weight (kg)	0.26 ± 0.00^a^	0.27 ± 0.01^a, b^	0.26 ± 0.01^a^	0.28 ± 0.01^b^	0.29 ± 0.00^b^
T.weight (g)	1.26 ± 0.04^a^	1.35 ± 0.04^a, c^	1.52 ± 0.06^b^	1.53 ± 0.02^b^	1.42 ± 0.07^b, c^
GSI (g/kg)	4.78 ± 0.43^a^	5.18 ± 0.15^a^	5.72 ± 0.36^a^	5.21 ± 0.08^a^	5.05 ± 0.46^a^
E.weight (g)	0.49 ± 0.03^a^	0.54 ± 0.01^a, b^	0.53 ± 0.01^a, b^	0.57 ± 0.05^a, b, c^	0.58 ± 0.01^c^
Tail weight (g)	0.18 ± 0.01^a^	0.18 ± 0.00^a^	0.19 ± 0.02^a^	0.19 ± 0.01^a^	0.19 ± 0.01^a^
ESI (g/kg)	1.99 ± 0.08^a^	2.13 ± 0.07^a^	1.88 ± 0.10^a^	1.99 ± 0.04^a^	2.05 ± 0.12^a^

Means ± SEM with different superscripts (a, b, c) in the same row differ significantly (*P* ≤ 0.05); identical superscripts indicate no significant difference (*P* > 0.05). GSI, gonadosomatic index; ESI, epididymosomatic index; T, testicular; E, epididymal.

#### 3.4.1 . Effect of nifedipine on mean rat body weights and the protective effects of vitamin C and garlic

Although groups D and E showed increased (*P* = 0.024 and 0.013, respectively) mean body weights compared to group A (control), the increases were not statistically different from group B (nifedipine group) at *P* > 0.05.

#### 3.4.2 . Effect of nifedipine on mean testicular weights and the protective effects of vitamin C and garlic

Group B showed an increase in testicular weight compared to A, however, the increase was not statistically significant (*P* = 0.225). On the other hand, groups C, D and E showed a significant increase in testicular weight compared to group A (*P* = 0.003, 0.002, and 0.041) and C and D alone showed significant increase compared to B (*P* = 0.032 and 0.027, respectively).

#### 3.4.3 . Effect of nifedipine on mean gonadosomatic index (GSI), epididymal weight, tail weight and epididymosomatic index (ESI) and the ameliorative effects of vitamin C and garlic

There was no statistical significance found in the mean GSI, tail weight and ESI across the groups. However, a significant increase in epididymal weight was observed in group E (Vitamin C + garlic) compared to group B (nifedipine) at *P* = 0.042.

## 4 Discussion

The present study evaluated the ameliorative effects of vitamin C and garlic on nifedipine-induced reproductive disorders. The findings revealed a trend toward improved reproductive efficiency in male albino rats treated with nifedipine in conjunction with natural supplements. Nifedipine is an effective antihypertensive agent (14); however, its adverse effects on male reproductive health have been widely reported. It interferes with spermatogenesis and other sperm quality indices (12). This study hypothesized that vitamin C and garlic, as potent exogenous antioxidants, could mitigate the reproductive toxicity associated with nifedipine administration.

### 4.1 Nifedipine-induced testicular, epididymal, and sperm morphological abnormalities

Drug-induced toxicity often compromises organ function by increasing reactive oxidants beyond the body's antioxidant capacity (46, 47). In this study, nifedipine administration caused severe testicular degeneration, consistent with established hallmarks of chemical-induced reproductive toxicity. The testis is responsible for sperm production through spermatogenesis, a tightly regulated process that begins with spermatogonia and progresses through spermatocytes, spermatids, and spermatozoa (48, 49). However, spermatozoa emerging from the rete testis are immature and non-motile; they acquire motility and fertilizing capacity within the epididymis (42).

Nifedipine exposure led to testicular degeneration, indicating compromised gonadal function (50). The observed depletion of spermatogenic cells signifies disruption of the germ-cell developmental hierarchy required for effective spermatogenesis (48–51). The reduced epithelial thickness suggests Sertoli cell damage, while vacuolation within the seminiferous epithelium indicates germ cell degeneration and impaired spermatogenic architecture.

Decreased tubular diameter observed in the treated groups indicates cellular shrinkage and loss of tubular content, leading to diminished sperm output. The interstitial compartment of the testes, comprising Leydig cells, connective tissue, immune cells, and blood vessels, is crucial for testosterone synthesis and nutrient exchange (52–54). The increased interstitial space in nifedipine-treated rats suggests fibrosis and oedema, which may reduce the number of functional Leydig cells and compromise testosterone production (52, 54).

These structural abnormalities collectively pose a serious risk to male fertility. While Iranloye et al. (17) reported minimal testicular alterations, other studies have shown pronounced negative impacts of nifedipine on sperm count, motility, and hormone levels (55). Discrepancies across studies may result from differences in dosage, exposure duration, species, or experimental conditions. Interestingly, Ruigrok et al. (56) reported a protective role of nifedipine in an ischemia/reperfusion model, highlighting its context-dependent biological effects.

The epididymis connects the efferent ductus to the vas deferens and is responsible for sperm concentration, maturation, storage, membrane remodeling, and structural stabilization (57). The severe epididymal degeneration observed in nifedipine-treated group may likely impair these functions, leading to reduced sperm quality and fertility potential. Thus, the structural lesions seen in the epididymal tissue provide a plausible explanation for the concurrent decline in sperm quality. Similar degenerative features, such as epithelial thinning, luminal sperm depletion, and vacuolation, have been reported in response to drug-induced toxicity (58). Epididymal epithelial cells are responsible for fluid reabsorption and secretion of factors vital for sperm maturation and motility (57, 59). Their loss, as observed here, could lead to ejaculation of immature, non-motile spermatozoa, consistent with the findings of Pan et al. (60), who linked epithelial injury to abnormal sperm maturation. The loss of connective tissue integrity compromises mechanical support, vascular supply, and immune protection (54). Decreased tubular diameter and distortion of the lumen suggest atrophy and potential obstruction of sperm transit (61).

Basement membrane thickening, also observed in this study, can impede nutrient and oxygen diffusion to the epithelium and is commonly associated with fibrotic changes (62, 63). The tubular fibrosis and interstitial expansion noted in this study cannot be mechanistically confirmed; however, earlier studies suggest that nifedipine induces oxidative stress (55). Oxidative stress is known to promote extracellular matrix (ECM) accumulation and fibrotic remodeling. ECM deposition may offer a plausible explanation for these histological changes, which may lead to structural rigidity and impaired sperm flow. Our findings align with previous reports linking oxidative stress–mediated fibrosis to reproductive toxicity (54, 61).

Nifedipine treatment significantly altered sperm morphology, producing defects such as coiled and bent tails, fractured necks, detached heads, and an overall reduction in normal sperm cells. These findings are consistent with with the report of Kanwar et al. (16), who observed similar structural disintegration and tail coiling in sperm exposed to nifedipine. The morphological abnormalities observed in the present study indicate structural compromise of spermatozoa, which may reflect impaired membrane integrity or cytoskeletal disruption. Although the precise mechanisms were not evaluated in this study, previous research has associated nifedipine exposure with cellular alterations that could contribute to such defects (64, 65).

### 4.2 . Ameliorative effect of vitamin C and garlic on testicular and epididymal degeneration, and sperm abnormalities

Our results demonstrated that vitamin C alone showed a trend toward improvement against nifedipine-induced testicular and epididymal damage than garlic alone or their combination. This pattern suggests that co-administration may not necessarily enhance their antioxidant effects. This agrees with numerous studies showing that vitamin C exerts potent antioxidant protection against testicular injury caused by toxins such as lead, pesticides, and chemotherapeutic agents (66–69). Similar observations have been reported, where individual antioxidants outperformed combinations, possibly due to differences in their cellular targets or redox interactions that influence overall antioxidant activity (70). Additionally, the present finding aligns with the observations of Afolabi et al. (71), who reported no additive benefit from combining antioxidant supplements.

Vitamin C's mechanism of protection is largely due to its water-soluble antioxidant capacity, allowing it to scavenge reactive oxygen species (ROS) and prevent lipid peroxidation of sperm membranes. Unlike classical synergistic antioxidant pairs such as vitamin C and vitamin E, which regenerate each other through inter-antioxidant recycling, vitamin C does not regenerate garlic's organosulfur antioxidants (redox cycling interference) (72, 73). Therefore, the combination may not enhance total antioxidant capacity as expected. Instead, both antioxidants may act on overlapping ROS and compete for redox cycling pathways (e.g., glutathione or NADPH systems), resulting in diminished efficacy compared to vitamin C alone. To further buttress this, antioxidants differ in the types of ROS they target, but some also act on the same radical species. For example, both vitamin C and garlic neutralize superoxide anions, which drives lipid peroxidation of sperm membranes, compromising membrane fluidity and sperm motility Wang et al. (74). Consequently, when both vitamin C and garlic primarily scavenge the same superoxide anion radicals, they end up competing for the same oxidative targets rather than complementing each other. This overlap may have made the combined treatment *appear weaker* than the individual treatments.

Conversely, neither Vitamin C (2.5% slight increase) nor garlic (0.25% slight increase) administered separately, fully restored normal sperm morphology. However, the combination treatment, significantly increased the proportion of morphologically normal sperm by 4% compared to the nifedipine group, suggesting a partial ameliorative effect. Vitamin C alone also produced a modest improvement by reducing the percentage of detached heads by 1.5%, indicating that vitamin C may have contributed more substantially to the observed improvements, even when co-administered with garlic. The comparatively better performance of vitamin C may be related to the distinct modes of action of the two antioxidants: garlic's organosulfur compounds tend to act more gradually by activating endogenous antioxidant systems such as the Nrf2 pathway (75), whereas vitamin C exerts a more immediate effect by directly scavenging reactive oxygen species and regenerating endogenous antioxidants (76). These mechanistic differences previously reported may help explain why vitamin C demonstrated slight improvement in the present study.

### 4.3 . Nifedipine-induced sperm functional impairments and ameliorative effects of the supplements

Nifedipine markedly impaired sperm motility (44.35% reduction relative to the control). Although antioxidant supplementation produced slight increases in motility in groups C and E (2.35% and 6.1%, respectively), these changes were not statistically significant, and the ameliorative effect was not pronounced. This suggests that persistent structural or metabolic dysfunction remained despite antioxidant treatment. Calcium channel blockade may directly inhibit ATPase activity and calcium uptake essential for flagellar motion, as described by Kanwar et al. (16), which may explain the limited recovery of motility in the treated groups.

Sperm concentrations in both testes (TSC) and epididymides (ESC) were also reduced, indicating suppressed spermatogenesis.A significant recovery was observed in antioxidant-treated groups, particularly in groups D and E, with epididymal sperm concentration (ESC) increasing by 0.40% and 0.46% and testicular sperm concentration (TSC) by 1.7% and 0.88%, respectively. The most notable improvement was recorded in the garlic-treated and the combination (vitamin C + garlic) groups, suggesting that garlic may have contributed more strongly to the observed recovery; however, the underlying mechanism remains unclear and warrants further investigation. Simillarly, the combination therapy (group E) slightly improved the viability of sperm cell by 3.15% increase, which was not statistically different from the control, suggesting some protective benefit. However, despite the significant 4% increase in normal sperm morphology, the sperm population may still contain morphologically normal but functionally compromised cells, explaining the limited motility recovery.

### 4.4 Ameliorative effects on somatic and organ weights

Unlike earlier studies that reported testicular atrophy following calcium channel blocker exposure (77), nifedipine-treated rats in this study showed a slight increase in testicular and epididymal weights. This paradoxical finding may likely reflect mild tissue oedema, congestion, or compensatory hypertrophy rather than functional improvement, as supported by reduced sperm indices and histological alterations. In contrast, the greater organ weights observed in the vitamin C and garlic-treated groups may be biologically meaningful because they paralleled increases in testicular sperm count (TSC) and epididymal sperm count (ESC). This pattern indicates restorative hypertrophy, where improved spermatogenic activity and enhanced epididymal sperm storage contribute to increased tissue mass. Thus, the weight gain in the antioxidant groups reflects functional recovery of the testes and epididymis, rather than the non-beneficial swelling seen with nifedipine alone.

## 5 Conclusions

This study demonstrates that nifedipine administration induces significant reproductive toxicity in male rats, characterized by testicular and epididymal degeneration and impaired sperm quality. Supplementation with vitamin C and garlic, individually or in combination, showed a trend toward mitigating these adverse effects, with vitamin C alone (group C) and its combination with garlic (group E) providing the most pronounced protective response. The findings highlight the therapeutic value of natural antioxidants in reducing drug-induced reproductive dysfunction and provide experimental support for their use in preserving reproductive health during chronic nifedipine therapy in pet animals and humans.

### 5.1 Study limitations

The study did not include biochemical assays of oxidative stress markers such as MDA, SOD, CAT, or GSH. Therefore, although the reproductive improvements observed in the antioxidant-treated groups are consistent with reduced oxidative stress, this mechanistic pathway remains inferred rather than directly confirmed, representing a limitation of the study.

Another limitation of this study is the sample size (*n* = 4), which may reduce the statistical power to detect subtle treatment differences. However, assumptions of normality and homogeneity of variance were examined and met for most continuous variables, supporting the use of parametric analyses.

Finally, although commercially prepared garlic powder was used, it was not standardized for specific bioactive constituents (e.g., allicin content). This lack of phytochemical standardization may influence reproducibility and makes comparisons with studies using quantified or purified garlic extracts more challenging.

## 6 Recommendations

- Future studies should include direct biochemical assessment of oxidative stress biomarkers such as malondialdehyde (MDA), superoxide dismutase (SOD), catalase (CAT), and glutathione (GSH) in testicular and epididymal homogenates to validate the redox mechanism in this model.
- The use of higher doses of vitamin C and garlic (300–400 mg/kg) should be explored to determine the optimal protective concentration and possible synergistic thresholds.
- Standardized garlic powder should be used in future studies.
- Future studies may incorporate a matched vehicle control to eliminate even minimal solvent-related influences.
- A suggestion to test combined antioxidant regimens with controlled dose ratios to confirm or refute antagonism.
- The inclusion of hormonal profiling in future work.
- Consideration of biochemical and molecular endpoints (e.g., Nrf2, Bcl-2, Caspase-3 expression).

## Data Availability

The original contributions presented in the study are included in the article/supplementary material, further inquiries can be directed to the corresponding authors.
